# A novel method for isolation of human lung T cells from lung resection tissue reveals increased expression of GAPDH and CXCR6

**DOI:** 10.1016/j.jim.2008.12.001

**Published:** 2009-03-15

**Authors:** C.E. Day, S.D. Zhang, J. Riley, T. Gant, A.J. Wardlaw, C. Guillen

**Affiliations:** a Institute for Lung Health, Department of Infection, Immunity and Inflammation, University of Leicester, Glenfield Hospital, Leicester LE3 9QP, United Kingdom; b MRC Toxicology Unit, Hodgkin Building, University of Leicester, PO Box 138, Lancaster Road, Leicester LE1 9HN, United Kingdom

**Keywords:** FBS, Fetal bovine serum, GAPDH, Glyceraldehyde 3-Phosphate Dehydrogenase, RT-PCR, Reverse transcription polymerase chain reaction, PI, Propidium Iodide, Lung, T lymphocytes, Alveolar macrophages, Flow cytometry, Microarray

## Abstract

Lung T lymphocytes are important in pulmonary immunity and inflammation. It has been difficult to study these cells due to contamination with other cell types, mainly alveolar macrophages. We have developed a novel method for isolating lung T cells from lung resection tissue, using a combination of approaches. Firstly the lung tissue was finely chopped and filtered through a nylon mesh. Lymphocytic cells were enriched by Percoll density centrifugation and the T cells purified using human CD3 microbeads, resulting in 90.5% ± 1.9% (*n* = 11) pure lymphocytes. The T cell yield from the crude cell preparation was 10.8 ± 2.1% and viability, calculated using propidium iodide (PI) staining and trypan blue, was typically over 95%. The purification process did not affect expression of CD69 or CD103, nor was there a difference in the proportion of CD4 and CD8 cells between the starting population and the purified cells. Microarray analysis and real time RT-PCR revealed upregulation of GAPDH and CXCR6 of the lung T cells as compared to blood-derived T cells. This technique highly enriches lung T cells to allow detailed investigation of the biology of these cells.

## 1 Introduction

The lung has a large mucosal surface area and is constantly exposed to a wide range of airborne antigens. As an important site of antigen entry, the adult lung is populated by large numbers of memory T cells which are thought to be important in lung host defence (Wardlaw and Hamid, 2002). T cells mainly reside within the bronchial lamina propria, in the alveolar walls and the interstitium (Campbell et al., 2001). There are more T cells in the lung than in the peripheral circulation and in a normal lung there have been calculated to be ten times more T cells than neutrophils (Pabst et al., 1987). Lung T cells are involved in initiating and regulating the immune response to pathogens, and the importance of lung CD4 T cells is demonstrated by the fact that AIDS patients are very susceptible to pathogens such as *Pnemocystis carinii*, which rarely infects individuals with an intact immune system (Rosen et al., 1985). However, lung T cells are also involved in inflammatory conditions affecting the lung, such as asthma (Brightling et al., 2002) and sarcoidosis (Inui et al., 2001). It would therefore be of great interest to characterise and study human lung T cells *ex vivo* and *in vitro*.

So far, it has been difficult to purify lung T cells due to contamination of other cell types, particularly alveolar macrophages. Not only are the alveolar macrophages in abundance, they also complicate the purification of T cells through their ability to ingest particles and adhere to surfaces. It is therefore difficult to deplete these cells without also decreasing the yield of lymphocytes. By a combination of approaches, we have developed a novel method for isolating human lung T cells from lung resection tissue from patients undergoing surgery for lung carcinoma. The phenotype of the purified cells was characterised with flow cytometry and by gene expression using gene array and quantitative reverse transcriptase polymerase chain reaction (RT-PCR).

## 2 Materials and methods

### 2.1 Lung filtrate

Lung resection tissue was obtained from patients undergoing surgery for carcinoma at the Glenfield Hospital in Leicester, UK. All patients gave written informed consent and the study was approved by the Leicestershire Research Ethics Committee. Macroscopically normal tissue was excised at least 2 cm from the tumour. The specimens usually weighed between 10 and 40 g.

### 2.2 Media and reagents

Media: RPMI-1640 (with L-glutamine and 25 mM Hepes) (Invitrogen, Paisley, UK) supplemented with 10% heat-inactivated fetal bovine serum (FBS) (Sigma, Dorset, UK) and antibiotics (Sigma). Histopaque 1077 was purchased from Sigma. The osmolarity of Percoll (Sigma) and media was adjusted to 285 mOsm with 10× phosphate buffered saline (PBS) (Invitrogen) or distilled water, respectively. Percoll was diluted in media to obtain the 40% and 70% Percoll solutions.

### 2.3 Purification protocol

The lung specimen was placed in media and finely chopped at room temperature using scissors. The cell suspension was then filtered twice through 100 μm gauze (Fisher Scientific, Loughborough, UK). The resulting filtrate was centrifuged for 10 min at + 4 °C, 1400 rpm (394 *g*), after which erythrocytes were lysed using ice cold distilled water for 30 s. An equal volume of ice cold 2× PBS was immediately added and the cells were washed once. The cell pellet was resuspended in 40% Percoll, carefully layered onto 70% Percoll and centrifuged at 2000 rpm (804 *g*), without brake, at + 4 °C for 30 min. After centrifugation, two layers of cells were formed. One upper layer on top of the gradient, containing mainly macrophages, and one lower, lymphocyte enriched, layer in the interface between the two Percoll concentrations. Each layer was carefully aspirated and washed twice in cold PBS. CD3+ cells were further purified by magnetic separation using anti-CD3 conjugated microbeads and LS columns (Miltenyi Biotech, Surrey UK), according to manufacturer's instructions. Finally, purity was assessed with Kimura stain. The Kimura counts were confirmed using Romanowsky stained cytospins. Viability was assessed by Trypan blue (Sigma) and propidium iodide (BD Biosciences, Oxford, UK).

### 2.4 Flow cytometry

For flow cytometry, the following antibodies were used: CD103-PE CD69 (Invitrogen), CD4-FITC and CD8-PE (BD) and secondary FITC-labelled rabbit anti mouse antibody (DAKO cytomation, Cambridgeshire, UK). FITC-conjugated Annexin V and propidium iodide (PI) were both from BD. All stained cells were analysed in a FACSCanto flow cytometer (BD), using FACSDiva software (BD).

### 2.5 Microarray

Two purified lung T cell preparations (83 million cells, 99% pure and 20 million cells, 97% pure, respectively) were compared to two blood T cell samples from healthy donors (10 million cells, 26 million cells, both > 95% pure), which were isolated using the same purification protocol. Total RNA was extracted using TRIzol Reagent (Invitrogen) according to the manufacturer's protocol. The lung T cell preparations yielded 11 μg and 6.7 μg of RNA respectively. From the blood samples, 8.5 μg and 8.7 μg of RNA was obtained.

Microarray experiments were performed using a 3DNA Array 350 kit from Genisphere (Hatfield, PA, USA) and microarray slides constructed in the MRC toxicology unit in Leicester, as previously described (Turton et al., 2001). The microarray slides contained 6000 genes from the human genome. Two samples (2.5 ug each) labelled with two different fluorescent dyes (eg the blood sample was labelled with Cy3 dye and the lung sample with Cy5 dye) were allowed to hybridize with each slide. For each pair of samples, two microarray slides were used with a reverse-labelling (dye swapping) design. Microarray slides were scanned using an Axon GenePix 4200A scanner (Axon Instruments, Union City, CA) to acquire the microarray images which were subsequently processed with GenePix Pro 5 (Axon Instruments), according to the manufacturer's recommended settings. The median fluorescent intensity of a feature spot on a microarray slide was used to represent the expression level of the corresponding gene. No background subtraction was taken as more evidences are suggesting this may be a better choice than subtracting background. For quality control, spots flagged as bad (low quality) by the GenePix software automatically, or manually by experimenter after visual inspection of the microarray image, were filtered out and excluded from further analysis, so were the spots that had lower fluorescent intensity than their surrounding background.

Each microarray was then normalized by globally shifting the mode of log ratio values to 0, implicitly making the assumption that on each microarray slide most genes are not differentially expressed between the two sample channels. We chose global normalization instead of local normalizations such as Lowess normalization because we carried out reverse-labelling of the same pair of samples in our experimental design, and this reverse-labelling procedure was designed to rectify the dye biases. By averaging the results from the two dye-swapped slides for the same pair of samples, we can eliminate the dye biases that may be present. To identify differentially expressed genes, the averaged results for each pair of samples of the two dye-swapped slides were used. All the genes were first sorted by the overall fluorescence intensity in descending order. Only genes with an average fluorescence intensity of 1000 or higher for at least one sample were further considered for identification of differential gene expression. These genes were then sorted by the absolute value of their log ratio in descending order, so that the genes which were regarded as most differentially expressed were on top of the ordered list.

### 2.6 Real time quantitative RT-PCR

To confirm the results obtained in from the microarray experiments, one of the lung samples used in the microarray was compared to three blood samples from healthy donors. Total RNA was extracted as for the microarray and treated with DNAse I (Invitrogen). Reverse transcription was performed using Omniscript RT kit (Qiagen, Crawley, West Sussex, UK), according to manufacturer's protocol. Real time quantitative RT-PCR reactions were performed in a Stratagene MX3000P thermal cycler system (Stratagene, Amsterdam, The Netherlands), using Brilliant SYBR Green Q PCR Master mix (Stratagene) and the following primers: CXCR6: Forward 5′-GGC CCA CCA GAA GCA TTT AC-3′, Reverse 5′-TTA AGG CAG GCC CTC AGG-3′; GAPDH: Forward 5′-AAT GGA AAT CCC ATC ACC ATC T-3′, Reverse 5′-CGC CCC ACT TGA TTT TGG; 18 s: Forward: 5′-CTT AGA GGG ACA AGT GGC G-3′, Reverse 5′-ACG CTG AGC CAG TCA GTG TA-3′.

The conditions for the PCR reactions were:

95 °C for 10 min followed by 40 cycles of 94 °C for 15 s, 58 °C for 30 s and 72 °C for 30 s.

### 2.7 Statistical analysis

Student's *t* test was used to compare differences between groups unless where otherwise stated.

## 3 Results

### 3.1 Histopaque does not separate lung lymphocytes from erythrocytes

Histopaque is a polysucrose/sodium diatrizoate gradient which is designed to purify mononuclear cells from whole blood, but has also been used for cells suspended in media to separate granulocytes from mononuclear cells in blood (Symon et al., 1994). Since the lung filtrate is a complex mixture of all loose particles from the lung homogenate, we originally used Histopaque in an attempt to deplete debris, dead cells and erythrocytes. However, while Histopaque is effective for purification of peripheral blood mononuclear cells from whole blood, we found that a large proportion of lymphocytic cells were found in the cell pellet after centrifugation (data not shown). Therefore, we concluded that Histopaque was not suitable for enrichment of mononuclear cells from lung filtrate. Erythrocytes were instead removed using hypertonic lysis.

### 3.2 Adherence to plastic does not deplete alveolar macrophages efficiently

Since the adherence to plastic in serum free media is widely used to deplete or enrich macrophages and monocytes (Helinski et al., 1988; Sporn et al., 1990), we initially used this technique to deplete the alveolar macrophages from the lung filtrate. However, after two 1 h adherence steps at 37 °C, we found that even though the number of macrophages decreased to some extent, the purity of lymphocytes did not increase, due to loss of these cells in the adherence steps (data not shown). We therefore did not incorporate an adherence step into our final protocol.

### 3.3 40/70% Percoll gradient enrich lymphocytes from lung filtrate

**Fig. 1:**
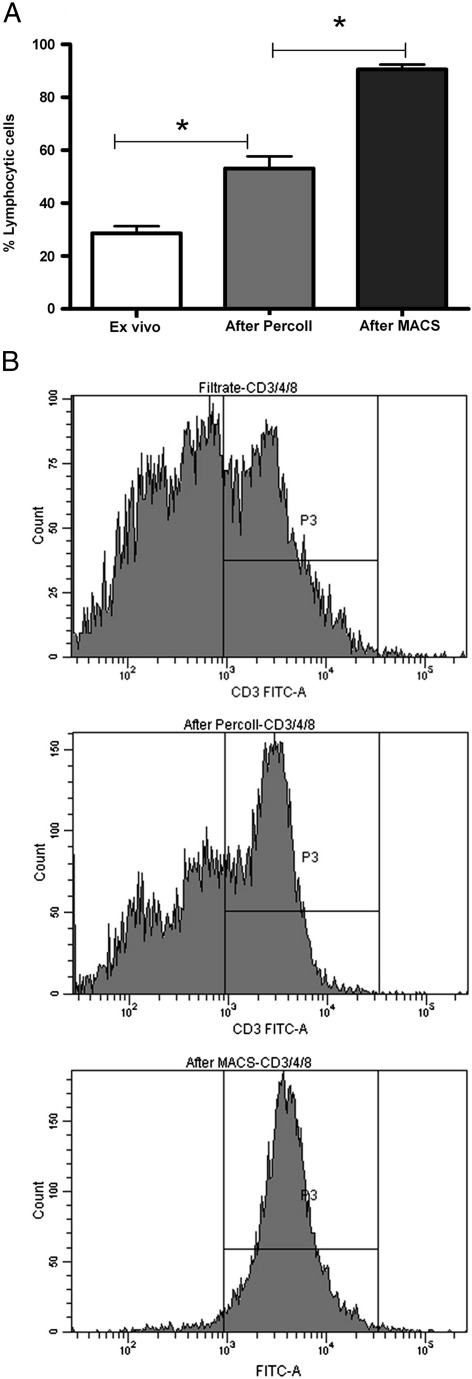
A: Purity of lymphocytic cells (as determined by Kimura stain) at different stages of the purification process. The filtrate contained 28.64 ±2.66% lymphocytic cells, which increased to 53.2 ± 4.56% (*p* < 0.0001) after Percoll gradient and to 90.5 ± 1.91 (*p* = 0.0013, *n* = 11) after purification with microbeads to CD3. B: Representative FACS histograms showing the expression of CD3, from the top: filtrate, lymphocyte layer after Percoll and after MACS purification.

Percoll is a modified silica gel that separates cells according to density and has been used to purify cell types from various tissues such as the liver and the gut (Svensson et al., 2002; Xu et al., 2002). Based on previous protocols (Svensson et al., 2002), we therefore used a discontinuous 40/70% Percoll gradient to separate macrophages from the lymphocytic cells in the lung filtrate. After centrifugation two layers were formed: A lower layer between the two Percoll concentrations which contained an enriched population of lymphocytes, whereas most alveolar macrophages and epithelial cells formed a layer on top of the gradient. The only cells which were pelleted at the bottom of the tube were erythrocytes. After Percoll density centrifugation, the purity of lymphocytic cells (by morphology) increased from 28.63 +/− 12.5% to 53.14 ± 21.5% (*n* = 22) (Fig. 1A). Flow cytometry analysis also showed a progressive increase in the percentage of CD3+ cells (Fig. 1B).

### 3.4 High purity and good yield of lung T cells using CD3 labelled microbeads

To isolate T cells from the lymphocyte enriched cell preparation, we used CD3-conjugated MACS beads, which purified the cells to 90.5 ± 1.9% (*n* = 11) (Fig. 1A). Previous purification with Percoll was required to decrease the contamination by alveolar macrophages, which otherwise block the columns. Using a more pure preparation for immunomagnetic separation also minimises the amount of beads needed, since this is calculated on the total number of cells. The yield, calculated as the number of purified CD3^+^ cells divided by the number of lymphocytic cells in the filtrate, was 10.8 ± 2.1 (*n* = 33) %. This figure is an underestimate, since the CD3^+^ cells only constitute approximately 60.62 ± 3.6% (*n* = 25) of all lymphocytic cells.

### 3.5 Phenotyping of purified lung T cells

The viability of the purified cells as determined by trypan blue and PI staining was typically over 95%. Staining the cells with FITC-conjugated Annexin V, which detects early apoptosis, showed that 18.53 ± 4.1% (*n* = 6) of the cells were apoptotic after purification. Before purification, 5.54 ± 0.97% (*n* = 4) of the cells bound Annexin V, which shows that apoptotic cells were already present in the cell preparation before the purification began although there was a modest increase in apoptosis as part of the purification procedure.

The purified lung T cells were phenotyped using flow cytometry to demonstrate that the purification did not alter the expression of activation markers or selected for certain T cell subsets. There was no difference in the ratio of CD8 and CD4 cells before and after purification (CD8:CD4 ratio was 1.298 ± 0.43 and 1.088 ± 0.24, respectively, *p* = 0.62, *n* = 5), neither did the purification process affect the expression of CD103 (36.78 ± 14.2% before purification and 27.50 ± 9.10% after purification, *p* = 0.29, *n* = 5) or CD69 (59.50 ± 15.9% before purification, 53.84 ± 13.1% after purification, *p* = 0.49, *n* = 5).

**Fig. 2:**
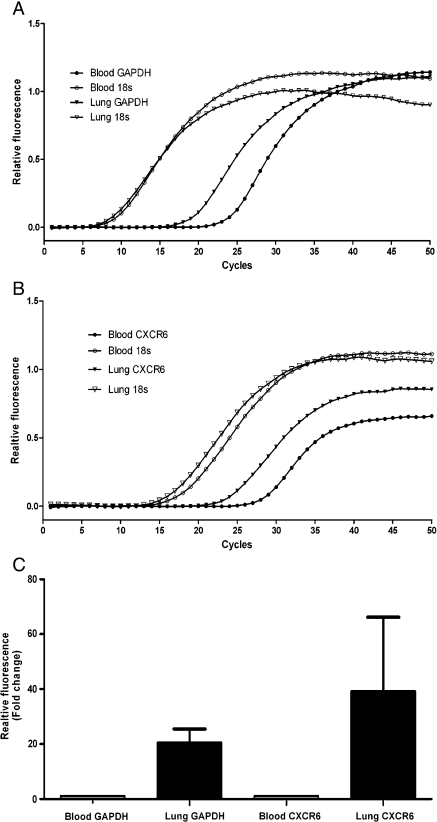
Representative Quantitative RT-PCR amplification plots of GAPDH (A) and CXCR6 (B). C: GAPDH expression was upregulated 20.5 ± 5.04 times, and CXCR6 expression 39.3 ± 26.9 times, on lung T cells compared to blood T cells (*n* = 3). 18 s rRNA was used as a normalising housekeeping gene.

In order to determine if the enriched lung T cells could be used to investigate their phenotype the gene expression profile of lung to blood CD3^+^ cells was compared using microarray*.* The 20 most up- or downregulated genes from one experiment are shown in Table 1. Gene expression from a second patient demonstrated a similar pattern. In both arrays the gene which was most upregulated in the lung T cells compared to the blood T cells was Glyceraldehyde 3-Phosphate Dehydrogenase (GAPDH). This result was confirmed using Quantitative RT-PCR, which demonstrated a 20.5 ± 5.04 times upregulation of GAPDH on lung T cells compared to blood T cells, whereas 18 s rRNA was expressed at very similar levels in the two cell types (Fig. 2). The upregulated genes also included CXCR6 which we have previously shown to be increased on lung compared to blood T cells, and L-selectin which is not expressed by lung T cells. We confirmed increased expression of CXCR6 using quantitative RT-PCR, which demonstrated a 39.3 ± 26.9 fold upregulation of CXCR6 on lung T cells compared to blood T cells (Fig. 2).

## 4 Discussion

The lung is a major portal of entry both for non-pathogenic antigenic material as well as a range of viral and bacterial pathogens. It therefore has a sophisticated immune system which involves barrier, innate and specific immune components. Investigation of lung T cells which make up an important part of lung immunity has been hampered by the difficulty in obtaining large numbers of relatively pure lung T cells.

As far as we are aware this is the first report that has focused on the purification of lymphocytes from human lung. In this paper we report a method that has allowed us to routinely obtain sufficient numbers of viable enriched lung-derived CD3 cells to undertake a detailed examination of their phenotype both by flow cytometry and gene expression.

Our source of lung T cells was resection tissue. Fine mincing of resected lung tissue results in the ‘falling out’ of large numbers of cells (mainly AM but with some lymphocytes and granulocytes). Enzymatically digestion of lung tissue (collagenase and hyaluronidase) released more lymphocytes from the lung tissue, but these cells were shown to have lost expression of certain chemokine receptors (data not shown). We can obtain about 10^7^ cells in total/gram of tissue from minced lung tissue although there is considerable variability in both the total number of cells and the percentage of lymphocytes depending on quality of the lung tissue. The receptor phenotype of these lung-derived T cells strongly suggests that they are resident in the lung tissue and not from the circulation as they are quite different from blood T cells in their expression of activation markers and have a similar profile of chemokine, adhesion and activation receptors to bronchoalveolar derived T cells (Campbell et al., 2001; Morgan et al., 2008). Lung T cells are found in a number of different compartments including the alveolar space samples by BAL, the bronchial epithelium and submucosa sampled by biopsy and the lung interstitium. The differences between the T cell phenotype in these compartments are not known although the similarities in receptor phenotype between BAL and lung resection derived cells are striking. Resection T cells are from the periphery of the lung and likely to be derived mainly from the interstitium. However there is no reason why our approach could not be used on BAL T cells although the number of cells obtained would be considerably less. The major problem with obtaining lung T cells has been the abundance of alveolar macrophages. Traditionally adherence to plastic has been used to deplete macrophage/monocyte populations but we found that equal numbers of lymphocytes adhered to the plates possibly due to the increased adhesion of lung compared to blood lymphocytes so that this step did not enrich the lymphocytes and we abandoned it. The lung filtrate contains a considerable amount of debris and we thought that a Histopaque step would remove red cells and debris. However this step resulted in loss of T cells and was also abandoned. We have in contrast found a discontinuous 40/70% Percoll gradient to be a reliable way to enrich the lymphocyte population. This allowed us to further enrich T cells using positive selection with immunomagnetic separation, which is a versatile approach as we could also enrich for other lymphocytic subsets if required. According to the manufacturer, the magnetic sorting does not result in downstream signalling or activation of the T cells. We did not detect down-regulation of CD3 after separation, as seen after activation with CD3/CD28 activating beads (data not shown).

The contaminating cells were mainly macrophages and if even greater purity was required an alternative would be to try and remove these by another method such as phagocytosis of iron filings. In microarray and PCR experiments, > 95% pure T cell preparations were used.

With any purification procedure there is the concern that one subset is being preferentially enriched. However at least in terms of CD4/CD8 and activation markers there was no difference in the starting versus the purified population. The lack of difference in CD69 expression which is rapidly upregulated with T cell activation also is some reassurance that the positive selection using an anti-CD3 antibody didn't activate the cells.

One of the aims of obtaining an enriched lung T cell population is to be able to do experiments which would not be possible with a filtrate sample. Flow cytometry can be done on unpurified lung T cells (although gating difficulties and background mean that this is not trivial) however studies involving gene expression need nearly pure populations. As a proof of concept that our methods resulted in a useful population of T cells we studied differences between unstimulated peripheral blood CD3 cells (enriched in identical fashion to the lung T cells) and the lung T cells using a customised gene array platform with a gene chip that contains only 6000 genes enriched for immune regulatory genes and most of the chemokine receptors We were gratified to observe that the differences in gene expression made sense in terms of what we know about the differences in receptor expression between the two populations. In particular we have previously shown that of all the chemokine receptors CXCR6 showed the most difference in expression between lung and blood T cells (Morgan et al., 2005). In the gene array it was striking that CXCR6 was the only chemokine receptor that showed up as being enriched on lung T cells and further supports the idea that this is a key receptor involved in constitutive migration of T cells to the lung. We confirmed this observation by quantitative RT-PCR on T cells from other donors. To our surprise the gene that came up as showing the most difference in gene expression was GAPDH, which is one of the key enzymes in glycolysis. We also confirmed increased expression by RT-PCR. GAPDH is often used as a housekeeping gene since it is constitutively expressed in most cell types. However, it has previously been shown that GAPDH is upregulated after activation in peripheral blood mononuclear cells and intestinal lymphocytes (Bas et al., 2004). Our results also suggest an upregulation of GAPDH in the lung T cells compared to the naive blood T cell. The mechanism for this and what it means in terms of the function of lung T cells remains to be investigated but it does suggest that GAPDH is not suitable as a house keeping gene for normalisation of mRNA expression levels in cells with different activation status. We did not compare blood and lung T cells from the same donor because of the logistical and ethical problems in obtaining blood from the patients undergoing surgery. The lung T cells were from patients undergoing lung resection whereas the blood T cells were from normal donors. We cannot exclude the possibility that our findings with GAPDH were not due to this difference although as the tissue was taken from relatively normal lung and the subjects were in good health with early stage lung cancer we feel it is unlikely. We have previously noted enriched CXCR6 expression on lung compared to blood T cells in matched donors so we think that this difference was undoubtedly independent of any donor effect. We therefore feel that the gene expression experiments have shown proof of principle that lung T cells purified according to the described method can be used to learn more about the function of these cells as well as raising intriguing questions about the glycolytic pathway in lung T cells and how this might relate to their function.

## Figures and Tables

**Table 1:** The list shows the 20 genes which were most differentially expressed in one of the two microarrays

Gene	Normalised log2 ratio of median
GAPD	1.74651
LDHA	1.6857
CD44	1.60579
CCL5	1.553
TNF-a	1.46178
HLA-DPB1	1.42163
L-selectin	− 1.37451
Ch6	1.34392
CREM	1.30224
PLP2	1.29657
ANXA1	1.29571
NR3C2	1.28638
PRG1	1.23003
FYN	1.22298
VIM	1.19836
CXCR6	1.16809
HLA-DRA	1.16795

Upregulation of expression compared to the control is expressed as the normalised log2 ratio of the median.
